# Pharmacogenomics Implementation and Hurdles to Overcome; In the Context of a Developing Country

**DOI:** 10.22037/ijpr.2021.114899.15091

**Published:** 2021

**Authors:** Nayyereh Ayati, Monireh Afzali, Mandana Hasanzad, Abbas Kebriaeezadeh, Ali Rajabzadeh, Shekoufeh Nikfar

**Affiliations:** a *Department of Pharmacoeconomics and Pharmaceutical Administration, Faculty of Pharmacy, Tehran University of Medical Sciences (TUMS), Tehran, Iran. *; b *Medical Genomics Research Center, Tehran Medical Sciences Branch, Islamic Azad University, Tehran, Iran. *; c *Personalized Medicine Research Center, Endocrinology and Metabolism Clinical Sciences Institute, Tehran University of Medical Sciences, Tehran, Iran. *; d *Department of Toxicology and Pharmacology, Faculty of Pharmacy, Tehran University of Medical Sciences (TUMS), Tehran, Iran. *; e *Department of Department of Industrial Management, Faculty of Management and Economics, Tarbiat Modares University, Tehran, Iran.*

**Keywords:** Pharmacogenomics, Precision medicine, Dynamic challenges, Policy note, Regulatory framework, Developing countries, Iran

## Abstract

Having multiple dimensions, uncertainties and several stakeholders, the costly pharmacogenomics (PGx) is associated with dynamic implementation complexities. Identification of these challenges is critical to harness its full potential, especially in developing countries with fragile healthcare systems and scarce resources. This is the first study aimed to identify most salient challenges related to PGx implementation, with respect to the experiences of early-adopters and local experts’ prospects, in the context of a developing country in the Middle East. To perform a comprehensive reconnaissance on PGx adoption challenges a scoping literature review was conducted based on national drug policy components: efficacy/safety, access, affordability and rational use of medicine (RUM). Strategic option development and analysis workshop method with cognitive mapping as the technique was used to evaluate challenges in the context of Iran. The cognitive maps were face-validated and analyzed via Decision Explorer XML. The findings indicated a complex network of issues relative to PGx adoption, categorized in national drug policy indicators. In the rational use of medicine category, ethics, education, bench -to- bedside strategies, guidelines, compliance, and health system issues were found. Clinical trial issues, test's utility, and biomarker validation were identified in the efficacy group. Affordability included pricing, reimbursement, and value assessment issues. Finally, access category included regulation, availability, and stakeholder management challenges. The current study identified the most significant challenges ahead of clinical implementation of PGx in a developing country. This could be the basis of a policy-note development in future work, which may consolidate vital communication among stakeholders and accelerate the efficient implementation in developing new-comer countries.

## Introduction

Precision medicine (PM) is an approach that changes medical practice from reactive to proactive by using genomics and other omics characteristics of patients. Pharmacogenomics (PGx), as an extension of pharmacogenetics, is the leading area in the field of PM and studies the interactions between genetics and pharmaceutical treatments by applying *in-vitro* companion diagnostics (CDx). CDx makes it possible to draw a line between biomarker availability and targeted therapy, which may not only significantly increase the probability of effectiveness and safety profile of pharmaceutical treatments (1, 2) but may potentially reduce the overall costs of therapy. PGx is generally considered a cost-effective strategy by improving health outcomes and depending on the prevalence of the condition, relevant biomarkers, and cost of CDxs (3, 4). Therefore, notwithstanding the high costs, there is a growing global interest in it; even in developing low- and middle-income countries (LMIC) with fragile healthcare delivery schemes, constrained resources and unstable economies (5-9). This includes Iran, which may benefit from PGx treatment strategies as a country with a considerable burden of non-communicable diseases and high levels of genetic diversity (10, 11).

Clinical implementation of PGx, although may improve a daunting variety of health issues in the developing world by efficient treatment of a range of conditions from infectious to non-communicable diseases, as it is associated with multiple uncertainties, stakeholders, and dimensions, it may lead to enormous dynamic implementation and access hurdles (7). This includes, and is not limited to, the readiness of healthcare systems fundamentally and financially, governance, access, reimbursement plans, awareness, and data infrastructures, as stated by studies in other developing countries (12, 13). 

 To avoid implementation inefficiencies, there is a vital need for evidence-based policy notes and operational frameworks which indicate relative challenges, consolidate the vital confidence among stakeholders and state the steps towards an efficient implementation. Since PGx research and adoption is at their early stages in Iran and to avoid wastage of limited resources due to inefficient implementation, it is vital to address main concerns based on early adopters experiences and stakeholders’ expectations. Identification of public-policy concerns is the first step in developing an evidence-based roadmap (14), which can later be used by relevant decision-makers. 

The current study aims to identify the most salient operational challenges related to the adoption of PGx in Iran concerning early adopters’ experience and experts’ expectations. This assessment is the first to address PGx implementation hurdles in the developing Middle-Eastern countries and could be the basis of the first evidence-based PGx policy-note and/or operational framework, in the future.

## Experimental

*Materials and Methods*

*Study Scope and Assessment Framework*

Current qualitative study investigated the implementation challenges of PGx based on early adopters’ experience and localized them for Iran as a developing country. It used an assessment framework including four national drug policy (NDP) components which are (1) efficacy/safety/quality, (2) access/availability, (3) affordability, and (4) rational use of medicine (15) as the main research question, which was answered using an interpretive approach.

Evidence synthetization was through a scoping review, which is one of the methods for broad and emerging matters where knowledge gaps and unclarified concepts lie (16, 17), including the topic of PGx (12). The study framework for the current scoping review was based on a 6-step methodology presented by Arksey *et al.* in 2005 (18) and Levac *et al.* in 2010 (19), and the objective was to examine the nature of the evidence on the questioned matter. As PROSPERO did not accept registrations for scoping reviews at the time of the study, the protocol was not registered; however, it was conducted based on a 27-item PRISMA-2009 checklist, which is the most comprehensive checklist for secondary studies (20). 

*Data Sources, Search Limitations, and Study Selections*

 The search was conducted by two independent reviewers on Medline (via PubMed), Embase, ISI, Scopus, and Google Scholar, as selected scientific databases. Data extraction was conducted through three steps of title, abstract and full-text screening. Exclusive keywords used were “Pharmacogenomics”, “Pharmacogenetics”, “efficacy, “safety”, “access”, availability”, “affordability”, and “rational use of medicine”. The eligibility criteria were any original or systematic review journal article published between 2003 (the year of human genome sequencing completion) to 1.1.2020, which was English, had accessible full text, and discussed PGx implementation challenges. All identified studies were imported into the EndNote Basic software. Complete search strategies (syntaxes) are available in Annex 1. A data charting form was developed by the researcher and used to extract the classified data from full texts of selected studies through a more narrative review. Numerical analysis was conducted on the results. Also, a qualitative analysis was conducted on the characteristics of the included studies. As the main goal was to adopt all possible challenges issued through a scoping review, no quality assessment was performed.

*Contextualization*

 To localize the categorized challenges in Iran, a focus group discussion with the strategic option development and analysis (SODA) workshop method was conducted. The reason for using SODA method and cognitive mapping technique was to better manage and extract the experts’ construct system on a dynamic matter which is associated with considerable uncertainties, multiple stakeholders, and many dimensions. This method was presented by Eden *et al.* in 2004 (21). Steps in this level were (1) information collection and primary elements assessment (PEA) through scoping review, which was explained in the previous step, (2) expert selection using list strategy, (3) focus group training with SODA workshop, (4) cognitive mapping and (5) data analysis.

For selecting experts, first, a list of experts was identified using the snowball selection method. In the snowball selection method, one person was selected initially, then recruited one additional subject, and the additional subject recruited another. Inclusion criteria were: 1) having a minimum of a PhD degree and 10-year relevant experience in at least one of the fields of genetics, pharmacogenomics, and Pharmacoeconomics, and pharmaceutical administration, 2) having relevant research on pharmacogenomics, and 3) having experience in an administrative or policy-making role at the pharmaceutical sector in the past five years. The list contained information including name, affiliation, phone number, and email of selected people. After preparing the list, 50% were picked randomly and were invited for the next steps by phone. Focus group training, using the SODA workshop method, was a two-hour pre-designed program in Tehran University of Medical Sciences which was an introductory to pharmacogenomics and the scoping review’s results. In the cognitive mapping step, ‘personal construct theory’ was practiced to capture personal construct systems and identify the expert’s values, beliefs and expectations of the dynamic issue at hand and use them to construct one aggregated system (21, 22). The cognitive maps were available to the subjects after the SODA workshop through email and was explained to them pre-completion through a one-hour meeting in Skype, individually. Data analysis was conducted with Decision Explorer XML (Demo Demonstration).

*Validation*

Face validation of semi-structured cognitive maps was conducted by two qualitative methodology experts, who were present in the pharmacogenomics SODA workshop.

## Results

**Figure 1:**
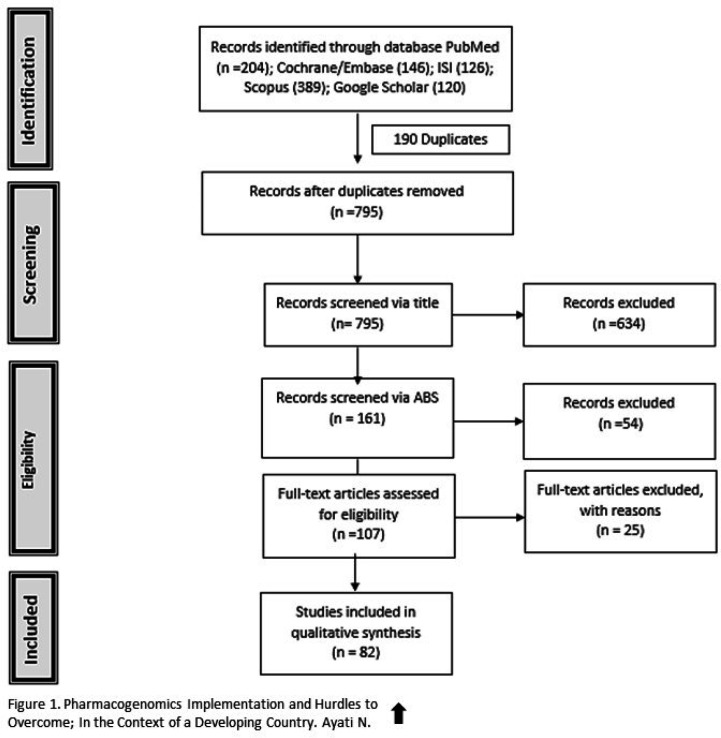
PRISMA Flowchart

*Scoping Review Results:* A scientific database search resulted in 985 articles (Embase: 146, Scopus: 389, ISI: 126, PubMed: 204, and Google Scholar: 120). Among them, 795 remained after duplication removal, and 161, 107, and 82 articles were selected through the title, abstract, and full-text screening, respectively (Figure 1). The final selected studies were published between 2002 to 2019, with 2013 having the most selected articles (n = 9, 11%). The majority of selected studies were conducted in The Unites States (n = 35, 42%) and Europe (n = 24, 29%, mainly Britain with n = 8, 9.75%). 

All included studies assessed the implementation and regulatory challenges as the main purpose but from different perspectives. As a result of the scoping review, 15 categorized predominant issues, sub-categorized in four national drug policy indicators, were identified as follows: 3 on efficacy/safety, 3 on access and availability, 3 on affordability, and 6 regarding the rational use of medicine (RUM). The recurrence of the challenges is summarized in Table 1.

*Contextualization of Results*

**Figure 2:**
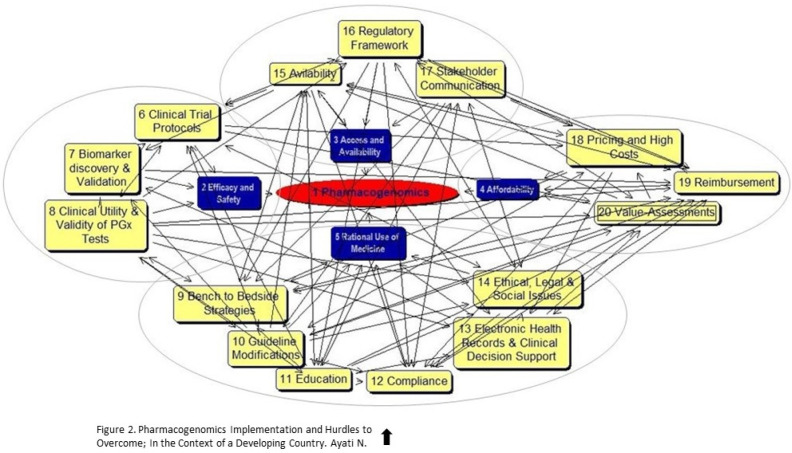
Collective cognitive map, indicating the multifaceted network of nodes and connections. Note: The map is not to be read. Dynamic challenges are centered around pharmacogenomics adoption, as the strategic goal. Main issues are shown in blue boxes; and sub-issues, clustered in gray circles are indicated in yellow boxes. PGx: pharmacogenomics

 PEAs were identified in the previous level and were used as the means of developing a semi-structured questioner, as the “personal construct theory” cognitive map. The snowball selection method identified 18 experts based on the inclusion criteria and list strategy. Among them, 8 were selected randomly. The male to female ratio of the participants were 1 and they had an average age of 48.6. All had a research history in the field of PGx (n = 8, 100%), three were clinicians with PhD degree and work/research experience in genetics, and PGx (n = 3, 37.5%) and five were pharmacists with PhD degrees in Pharmacoeconomics and pharmaceutical administration and had work experience in regulatory and policy-making roles in Iran’s FDA (n = 5, 62.5%). All participants participated in a 2-hour SODA training workshop, and a 1-hour individual session, and 7 (87.5%) completed the cognitive maps within the pre-defined timeline. The cumulated cognitive map is presented in Figure 2 with descriptive statistics in Table 2. These difficult-to-read kinds of maps, which include a complex network of node-labels and their connections, indicate the overall structure of the dynamic and complex problem and provide insight into it. 

## Discussion

Overall, a growing interest regarding PGx was recognized globally and locally among various stakeholders. However, regarding the existence of potential dynamic challenges and lack of operational frameworks addressing them, PGx adoption is disorganized and slow, especially in fragile healthcare systems in LMIC developing countries.

The results of this study identified the most salient issues facing PGx adoption and presented a complex network of dynamic challenges, which requires fundamental planning to overcome to be established effectively. These challenges, which were identified through a qualitative approach, were categorized based on national drug policy components and are discussed below. The discussion order is based on the iteration of the challenges in the scoping review. 

*Rational use of medicine*

 Irrational use of medicine is characterized mainly in inefficient adoption of treatment approaches, and there are proven strategies to promote it, including continuing education, supervision, practical clinical guidelines, compulsory regulations, the existence of an essential drug list, and ethics. This category had the most iteration number of challenges and is included below:

*Ethical, legal, and social issues*

ELSIs were associated with the highest number of repetitions among all and were indicated to be amongst the most important challenges of PGx adoption in Iran. Studies showed that the main challenges in this field are autonomy, health-technology access disparities, use of special populations in research, and the risk of racial geneticization in pharmaceutical development (23-27). Access equity issues, especially when making the treatment only available to the strongest responded patients on social expenses, was another challenge (27, 28). This may also lead to more diversities between the developed comparing developing countries (27). In addition, some studies issued ethical concerns regarding PGx clinical trial designs, which emphasizes knowledgeable consents, the economic value from the societal perspective, the generalizability of results, selected population, use of genetically altered animals for pharming, adequate oversight to justify a human trial and recruitment justice (27, 29, 30). 

*Education, for healthcare providers, regulators, patients, and the public*

The odds of irrational use of medicine may increase when introducing a new treatment strategy. Studies indicated that inadequate education of healthcare providers might appear as a barrier for efficient implementation of clinical PGx (31-37). Lack of education may also lead to irrational use of PGx-related testing (12, 38-40). A 2019 global survey on the progress of PGx education in medicine and pharmacy schools indicated that it is being seen as a necessity in a majority of study programs, and it has considerably improved in the last decade (41). It was indicated that public engagement and education may result in acceptance and adherence. Although this may be associated with some administrative challenges, education is a vital element in the transformation cycle and may enhance the effective implementation of PGx (42, 43). 

*PGx/CDx adherence and compliance*

 these were indicated in PEAs and addressed in two categories in the cognitive maps: healthcare providers and patients. Treatment adherence, as an effective factor in RUM, is defined as “the extent to which a person’s behavior corresponds with agreed recommendations from a healthcare provider” (44). A systematic review published in 2019 identified factors associated with non-adherence to pharmacotherapy among the patient population, naming socioeconomic status and education as effective factors (45). Educating patients and the general public on PGx and precision medicine principalsprinciples, will elevate the efficiency of PGx implementation and lead to treatment adherence (42). In addition, studies suggest that PGx testing itself may enhance adherence by reducing the concern of patients regarding the effectiveness and safety of a treatment strategy. It satisfies the patient by active participation in the treatment selection based on the test results and finally reducing the financial burden of trial and error (46-49). As for point of view of healthcare providers, a study showed that if physicians had comprehensive knowledge regarding PGx, molecular interpretation and test logistics (availability, cost, and coverage plans), it would increase their self-efficiency and enhance the implementation of PGx-testing (50). 

*Guideline development or modifications*

 Knowledge gaps and inadequate available information regarding PGx and test application is a concern of many clinicians, mainly due to the lack of comprehensive guidelines and also health information technology infrastructures (50-54). Studies suggest that the low rate of PGx implementation in clinical practice is due to the absence of transparent guidelines which can connect test results to clinical practice; therefore, some research networks such as the Clinical Pharmacogenetics Implementation Consortium (CPIC) provided clinical guidelines to facilitate the bench to bedside transformation process for PGx (55, 56). 

*Electronic health records and clinical decision support*

 In order for a physician to commit to RUM principles when applying PGx information in clinical practice, precise and up to date information is necessary in terms of electronic health records (EHR), to rely on (55). This may work through three main mechanisms: retrospective valuation of information in clinical settings, novel associations in real-world cohorts, and finally, real-time clinical decision support (CDS) (57). CDS is recognized as a viable tool when the efficient implementation of PGx is in question. The development of a patient-centric CDS, which can collect, interpret and translate data to practical information, may perform as an efficient approach (58). It has been successfully applied in some settings (58-62); however, it is still a global and local need.

*Bench to bedside strategies*

Implementation of PGx test results in clinical practice is associated with overcoming all operational challenges and is a multi-disciplinary and multi-stakeholder scientific, cultural, and political act (53, 63-65). However, from the clinical-practice perspective, the most important factor is the availability of robust genomic data, which associates the test results to the right medicine. This information mainly comes from expensive clinical trials that show rare genetic variants and some may be subjected to lack of generalizability (66). In addition, as mentioned above, the absence of agreed-upon clinical guidelines, CDS tools, regulatory issues, education, and compliance may be barriers to transforming PGx from bench to bedside (55, 62, 64, 67, 68). To accelerate this process, CPIC had suggested standardization acts on three main areas: clinical laboratory regulations, outcomes reporting and coverage. These, alongside integrated EHR, would enhance both medical practice and patient care (69).

*Efficacy/safety/quality-related challenges*

 Efficacy and safety are the first pillars to every drug policy. The current scoping review identified the following efficacy/safety-related challenges to be the main dynamic issues ahead of PGx and are discussed below:

*Clinical trials’ protocols*

 Efficacy and safety of personalized therapies are evaluated through clinical trials on biomarker-selected patients (70). Efficient adaptation of clinical trial protocols for evaluation of PGx medicinal products was one of the main stated challenges (70-73); however, as the pharmaceutical market in Iran is generic-based, not many clinical trials are conducted locally. Therefore, this challenge was identified as less important in Iran’s context based on the focus group opinion.

*Biomarker discovery and validation*

The identification and validation of Biomarkers is the key to clinical drug development and, accordingly efficient implementation of pharmacogenomics. However, their value depends on clinical utility and can be transformed into clinical use (74, 75). Despite advances in biomarker research, the translation of biomarkers into approved CDx is relatively low (76). This was identified as a challenge in the scoping review but again had minor importance in the context of Iran.

*Clinical utility and validity of test results*

Clinical utility of pharmacogenomics testing could be seen in prognostic and predictive test values. Data gaps in basic science, validity of biomarker translation, and lack of technological testing conditions may affect the clinical utility of pharmacogenomics testing. Accordingly, validation, scoring criteria, assay protocols, preservation methods, bio-specimen type selection, variation in patients’ drug regimens, and reporting quality may cause scientific and implementation challenges in PGx testing (75).

*Access*

Given the rising prices and constantly altering market, equitable access to quality health goods is a global multidimensional and multi-stakeholder challenge. According to WHO 2019-2023 roadmap on access to health products, there are two interlinked strategic components to access: equity improvement and efficiency assurance. This may be challenged due to scarce resources and unclear policy frameworks, among other barriers. As for PGx, equal access to effective medicines and CDxs is essential; however, some challenges such as lack of comprehensive frameworks, availability, stakeholder communication, affordability issues, and ELSIs may challenge access. 

*Regulatory frameworks*

As pharmaceuticals directly affect the lives of humans, governments need to ensure that the development, access, and use of medicines is regulated. This includes research/development, manufacturing, distribution, promotion/advertisement, marketing, import/export, supply chain, market inspection, drug labeling, and pharmacovigilance (77-79). The main hurdle facing regulatory agencies was the need for the regulatory schemes to adopt the new PGx/CDx requirements (80). Although there are different regulatory operational frameworks for PGx medicinal products and CDxs (81, 82), there are integrated ones which present linked legislation for PGx medicinal products and CDxs focusing on resolving pre-defined challenges in a distinct context or country (83-86). There is currently no PGx regulation framework in Iran; however, this study aims to be the first step in developing such a structure.

*Availability*

A handful of studies discussed the availability challenges for genomics-based pharmaceuticals and companion diagnostics (83-85, 87-92). However, based on the contribution from experts, availability is a considerable challenge in Iran. This is due to approval and the registration time-gap between the US and Iran’s FDA, for both pharmaceuticals and CDxs. By April 2020, there were 280 PGx-labeled FDA-approved pharmaceuticals. Among them, 122 (43.5%) were available on Iran’s drug list, which gained approval with a mean time gap of 12.36 years (99% confidence interval: 1-59, Annex 2) (93). This increases when CDxs are concerned; mainly due to the lack of co-development or drug-test registration time-gaps (94-96). As there is no governmental list of registered diagnostics in Iran, availability and registration time gap cannot be estimated. In addition, although some of CDxs are available in Iran, they are not available in core primary care centers in all cities and will challenge patient access. Co-approval of CDxs may increase their availability (89).

*Stakeholder communication*

 Implementation of PGx is a dynamic issue that requires communication between stakeholders; who may have different views and levels of awareness (12). Multiple stakeholders are associated with PGx implementation in Iran, naming: healthcare providers, patients, regulators, clinical associations, payers, investors, and the government as the owner. Some studies discuss the involvement of different stakeholders and their alliance in the process of an effective translation and implementation (83, 97). Among them, there is emphasis on the engagement of the general public, as the core beneficiary and the primary funder, and discovering their expectations and perception towards PGx (83, 98, 99).

*Affordability*

Implementation of pharmacogenomics and applying tests in a specific condition is highly dependent on whether the total value is higher than the total costs in a given setting with limited resources (100). Issues included in this category are discussed below:

*Pricing and high costs*

In the pharmaceutical and the diagnostics industries, which both have innovative and fast-growing markets, the revenue of goods is required to be in balance with the R&D costs and also the other value associated with that good; however, since the value may change based on time and context, the value-based price needs to be flexible (101). Value-based pricing is currently a well-known pricing method for pharmaceuticals, but diagnostics are mostly priced based on the cost-plus method and therefore may have low prices (40). Lower prices, especially in niche markets, lead to market negligence on some disease areas and harvests unmet needs. In some countries, the government incentivizes innovation in these areas by fast-tracking approvals, tax exemptions, grants, or market exclusivity (102, 103). 

*Reimbursement*

 Reimbursement supports patient access to novel health goods, especially those with high costs such as medicines and CDxs. In recent years, this financial contribution shifted towards outcome-based and risk-sharing payment models. These models are beneficial for manufacturers who can demonstrate the comparative value of their products and enable them to acquire extensive coverage and payer acceptance (104, 105). However, this requires strong administrative support and a follow-up database, which is not available in some countries, including Iran. In addition, the value of the companion diagnostics is not fully appreciated by health systems, and their market access framework is not fully aligned with drug-test co-reimbursement plans; therefore, patient affordability and eventually access would be affected (90, 106). From the payer perspective, although there are upsides to diagnostic coverage, including cost avoidance for non-responders and enhanced patient outcomes, there are also risks due to increased expenses for false results, an expanded patient population, and budget increases (104). In addition, fundamental ethical issues may appear when utilizing the results of Pharmacoeconomics studies for budget allocation decisions (107). A comprehensive review of the coverage policies of early adopters, for both PGx-medicines and CDx, is crucial for a developing country with scarce financial resources. Currently, in Iran, although some PGx-medicines are covered by insurance, no CDx is reimbursed by payers.

*Health Technology Assessments*

 When appraising the value of health technologies, both scientific and social values should be assessed during the early stages of drug development as the clinical effectiveness alone is not sufficient for the value judgment (108). The comparative economic evaluation for PGx and CDx (as a tool) lies on value demonstration in terms of incremental net health benefit and/or cost-effectiveness ratio, based on decision-makers’ objectives. This economic evaluation is associated with methodological, parameter, and structural uncertainties (109). It also may be subjected to issues regarding preciseness of the study question, identifying the place of intervention in the clinical pathway, data availability, and physicians’ compliance to uptake CDx (3, 110, 111). CDx may reduce patient-level heterogeneity, and cost/consequence of this reduction must be considered in the economic evaluations (109).

In addition to cost-effectiveness analysis, a budget impact analysis should be conducted for PGx-medicines and CDxs. It encompasses considerable financial burdens on healthcare systems and continued increases of the concerns on the sustainability of equitable and affordable access (12). This is important, especially in developing countries with very limited healthcare resources. In Iran, HTAs are mandatory for the Iran drug list and insurance basic list entry; however, it is not the routine for companion diagnostics registration or coverage.

The current study’s method was similar to some other studies (12, 14, 112). Chong HY *et al.* used scoping review and main stakeholder interviews to identify the landscape and the challenges of the PM adoption in the context of Southeast Asia. In this analysis, the assessment framework was based on a developed theme with six key indicators of health system, governance, access, awareness, implementation and data; while in the current study barriers were categorized based on NDP components. Chong H. Y. *et al.* study identified potential hazard for health disparities, lack of awareness, and political/financial supports to be the most prominent barriers for efficient implementation (12). Bashir NS *et al.* study, which was conducted to reach a policy framework on PGx testing in Canada, used scoping review to identify all the relevant barriers. These barriers were genetic discrimination, clinical trials, privacy, knowledge, stakeholder roles and clinical utility of tests. Also, it further utilized a policy development framework (3-I) to develop PGx policy. In contrast to the current, this study did not use a contextualization method to localize the results (14). Rafi I *et al.* used a qualitative study, including semi-structured interviews, to identify opportunities and barriers of PGx implementation into the United Kingdom clinical practice. In this study, which only used interviews as the source of data, interviewees were clinicians; however, the SODA workshop and individual cognitive maps were completed by multiple stakeholders in different sectors. The results of the Rafi *et al.* study showed that cost-effectiveness, availability, and ELSI of using genetic information in the primary care were the most salient issues (112). 

The current study is subjected to a few but important limitations; first, based on the broadness of the topic and due to the scoping review method (18, 19), quality assessment was not conducted on the selected studies. Second, the contextualization step and expert selection were conducted through the snowball approach, it may have been associated with selection bias, despite the random list strategy. However, as all participated experts were researchers experienced in health policymaking or pharmaceutical regulations, and also with adequate knowledge regarding PGx, it is believed that the significant implementation challenges are captured. 

**Table 1:** Distribution of identified challenges, sub-categorized based on NDP components

**Category**	**Issue**	**Recurrence (N)**	**Recurrence (%)**
Rational Use of Medicine (n = 103, 40.23%)	Ethical, legal and social issues	28	10.94
	Efficient bench to bedside strategies	14	5.47
	Guideline modifications	12	4.69
	compliance	7	2.53
	Electronic health records	5	1.95
	Education*	37	14.4
Efficacy/ Safety (n = 60, 23.44%)	Clinical trial designs and modifications	21	8.2
	Clinical utility, validity and reliability of associated tests	33	12.89
	Biomarker discovery and validation	6	2.34
Affordability (n = 52, 20.31%)	Pricing and high costs	21	8.2
	Reimbursement	16	6.25
	Health technology assessments	15	5.86
Access (n = 41, 16.02%)	Regulation (drug-test)	23	8.98
	Availability	11	4.3
	Stakeholder management	7	2.73

^*^Among education-related challenges, 6.69% were related to the education of healthcare providers, 8.1% to regulators and 4.58% to the patient/public.

**Table 2:** Descriptive statistics of the pharmacogenomics’ dynamic issues cognitive map

**Measure**	**Number in the cognitive map**
Concepts (nodes)	20
Links	114
Heads	1
Tails	0
Strategic center(s)	1
Major (main) dynamic issues	4
Minor (sub-) dynamic issues	15
Central concepts > 10 links	20*
Clusters	4
Loops	356

^*^All concepts were central and included more than 10 links, with reimbursement and regulatory framework

(16 links) being the most central and biomarker validation and discovery as the least (12 links).

## Conclusion

Despite the growing interest in the novel PGx era, even in developing countries with scarce resources, not enough is known about the possible hurdles facing efficient and evidence-based adoption of PGx to harness its full potential. In the current study, more salient challenges were identified through the experience of early adopters and the expectations of experts. The complex network of dynamic issues identified in the current study could be set as the matter of precedence and be the basis of the first pharmacogenomics policy note in the future. Although this study was conducted in the context of Iran, the findings could be extrapolated and used by developing middle-eastern countries where emerging interest in PGx adoption is expected. 

*Future Perspective*

There is an increasing interest in Pharmacogenomics globally, including developing low-middle-income countries that have limited budgets. Therefore, it is predicted that in a few years, drug research and also treatment approach will shift towards pharmacogenomics. This, makes it more vital to have updating pre-existing roadmaps and policy notes to harness this shift’s full potential.

## Conflict of Interests

The authors declare no conflict of interest.

## Author Contributions

All authors were involved in the conception and design of the study, collection of the data, analysis, and interpretation of the data, drafting of the article, and the provision of final approval of the article for submission/publication in the current journal. All authors agree to be accountable for all aspects of this work.
